# Genome Sequences of *Synechococcus* sp. Strain MIT S9220 and Cocultured Cyanophage SynMITS9220M01

**DOI:** 10.1128/MRA.00481-20

**Published:** 2020-07-23

**Authors:** B. Shafer Belisle, Andres A. Avila Paz, Angelina R. Carpenter, Tayla C. Cormier, Adam J. Lewis, Linnea S. Menin, Daniel R. Oliveira, BuKyung Song, Amy Szeto, Elizabeth I. Tchantouridze, Kayleigh A. Watson, Mary T. Yohannes, Nathan A. Ahlgren

**Affiliations:** a Biology Department, Clark University, Worcester, Massachusetts, USA; Loyola University Chicago

## Abstract

*Synechococcus* bacteria are unicellular cyanobacteria that contribute significantly to global marine primary production. We report the nearly complete genome sequence of *Synechococcus* sp. strain MIT S9220, which lacks the nitrate utilization genes present in most marine *Synechococcus* genomes. Assembly also produced the complete genome sequence of a cyanophage present in the MIT S9220 culture.

## ANNOUNCEMENT

Marine *Synechococcus* bacteria are globally distributed picocyanobacteria that contribute to ∼17% of annual net marine primary production (1). The genome of *Synechococcus* sp. strain MIT S9220, reported here, adds to the existing six isolate genomes from the CRD1 ecotype, which exhibits physiological and genomic adaptations to its low-Fe niche (2, 3). MIT S9220 was isolated from the equatorial Pacific (4) and cannot grow on nitrate, in contrast to most other marine *Synechococcus* species (4, 5).

DNA was extracted with a phenol-chloroform protocol (6, 7) from a late-exponential-phase, nonaxenic culture of MIT S9220 (obtained from Gabrielle Rocap), propagated in Pro99 seawater-based medium (8) at 23°C with 14:10-h light/dark illumination. Illumina sequencing (Nextera library kit; NextSeq 550) yielded 20,222,448 paired 150-bp reads, and Oxford Nanopore MinIon sequencing (library kit SQK-RAD004; R9.4.1 flow cell) produced 53,387 reads (60 Mb; *N*_50_, 1,727 bp). The Illumina reads were quality filtered with Trimmomatic v. 0.38 (9) with the following settings: ILLUMINACLIP:TruSeq3-PE-2.fa:2:30:10, LEADING:10, TRAILING:10, SLIDINGWINDOW:4:15, and MINLEN:50. Assembly with Unicycler v. 0.4.7 (10) with default settings using both Illumina and Nanopore reads yielded 13 contigs, 12 of which belong to MIT S9220 (2,406,645 total bp; *N*_50_, 1,568,415 bp; GC content, 56.4%; 300× mean coverage) based on high similarity to other CRD1 contigs (blastn; E value, <1e-84; identity, ≥88%). The MIT S9220 genome was annotated with the Prokaryotic Genome Annotation Pipeline (PGAP) v. 4.11 (11), and it contains 2,575 protein-coding genes, 42 tRNAs, and 2 rRNA operons. We estimate that the genome is >99% complete based on it possessing 1,026 homologs (reciprocal best hits; blastp; E value, <1e-10) of 1,033 single-copy core genes shared among *Synechococcus* isolate genomes (3, 12). Like other CRD1 genomes, MIT S9220 possesses a larger repertoire of Fe-related genes than most other *Synechococcus* genomes (3).

**FIG 1:**
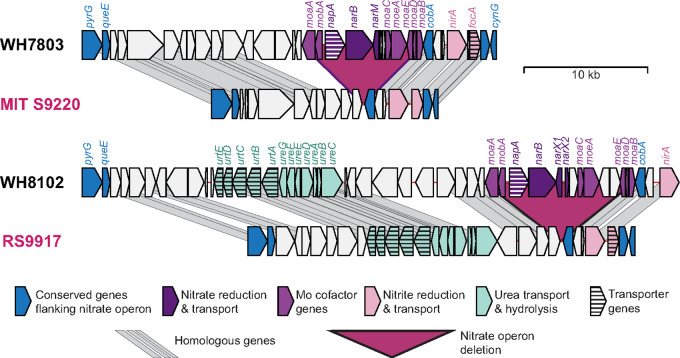
Regions in the MIT S9220 and RS9917 genomes (names in purple) that exhibit the loss of nitrate utilization genes (purple trapezoids) found in all other marine *Synechococcus* genomes sequenced to date. The nitrate operon includes genes encoding nitrate reductase (*narB*), a nitrate transporter (*napA*), and genes for synthesis of Mo cofactor required for nitrate reduction (*moaA* to *moaE*, *mobA*, and *moeA*). For reference, homologous regions are shown for the genomes of WH7803 and WH8102, which both have the nitrate utilization operon and either lack or possess, respectively, urea utilization genes (*urtA* to *urtE* and *ureA* to *ureG*) near the nitrate operon. Also labeled are the nitrate reduction (*nirA*) and transport (*focA*) genes and several conserved genes that flank the nitrate operon (*pyrG*, *queE*, *cobA*, and *cynG*).

MIT S9220 lacks the nitrate utilization genes found in most other marine *Synechococcus* species (Fig. 1) (13). A similar, parallel loss (3) occurs in *Synechococcus* sp. RS9917 (ecotype VIII) (Fig. 1) (14). MIT S9220 provides a valuable resource for further exploring the evolution of nitrogen utilization in these important phytoplankton, especially given similar loss and occasional reacquisition of these genes in closely related *Prochlorococcus* species (15, 16).

The remaining circular, 190,237-bp contig (38.8% GC content, 150× coverage), named SynMITS9220M01, is a *Caudovirales* T4-like myovirus based on high similarity to other cyanophage (top blastn results against the NCBI nonredundant [nr] database; E value, 0). SynMITS9220M01 could be an extrachromosomal phage, but it more likely represents an unintentionally cocultured phage obtained from unautoclaved, 0.2-μm-filtered Sargasso Sea water used for culture medium for several months during its propagation. SynMITS9220M01 is most similar to cyanophage S-SM2 (17) (average nucleotide identity [ANI], 72.3%). SynMITS9220M01 was annotated with Prokka v. 1.13.3 with default settings except for the option “–protein” used with a database of 325 marine cyanophage genomes. SynMITS9220M01 has 10 tRNAs and 239 predicted protein-coding genes, including the following host-acquired genes: phosphate-related genes (*phoH*, *pstS*) (17–20), photosynthesis genes (*psbA*, *psbD*, *petE*) (18, 21, 22), and others, such as *talC*, *mazG* (23–25), and genes involved in heptose-related lipopolysaccharide synthesis (d,d-heptose 7-phosphate kinase, phosphoheptose isomerase, and ADP-l-glycero-d-mannoheptose-6-epimerase) (26). SynMITS9220M01 adds to the growing database of >300 marine cyanophage genomes (27) and to our knowledge of cyanophage pangenomic diversity.

### Data availability.

*Synechococcus* sp. strain MIT S9220 is available from the Roscoff Culture Collection (http://roscoff-culture-collection.org/; strain RCC2571) or from Nathan A. Ahlgren upon request. Sequence data are available at NCBI under BioProject number PRJNA623799, including the raw data (accession numbers SRX8340787 and SRX8330445) and the assembled genomes (accession numbers JABBNJ000000000 and MT408532).
